# A Determination and Comparison of Urease Activity in Feces and Fresh Manure from Pig and Cattle in Relation to Ammonia Production and pH Changes

**DOI:** 10.1371/journal.pone.0110402

**Published:** 2014-11-14

**Authors:** Xiaorong Dai, Henrik Karring

**Affiliations:** Department of Chemical Engineering, Biotechnology and Environmental Technology, University of Southern Denmark, Odense, Denmark; National Institute of Agronomic Research, France

## Abstract

Ammonia emission from animal production is a major environmental problem and has impacts on the animal health and working environment inside production houses. Ammonia is formed in manure by the enzymatic degradation of urinary urea and catalyzed by urease that is present in feces. We have determined and compared the urease activity in feces and manure (a urine and feces mixture) from pigs and cattle at 25°C by using Michaelis-Menten kinetics. To obtain accurate estimates of kinetic parameters V_max_ and K'_m_, we used a 5 min reaction time to determine the initial reaction velocities based on total ammoniacal nitrogen (TAN) concentrations. The resulting V_max_ value (mmol urea hydrolyzed per kg wet feces per min) was 2.06±0.08 mmol urea/kg/min and 0.80±0.04 mmol urea/kg/min for pig feces and cattle feces, respectively. The K'_m_ values were 32.59±5.65 mmol urea/l and 15.43±2.94 mmol urea/l for pig feces and cattle feces, respectively. Thus, our results reveal that both the V_max_ and K'_m_ values of the urease activity for pig feces are more than 2-fold higher than those for cattle feces. The difference in urea hydrolysis rates between animal species is even more significant in fresh manure. The initial velocities of TAN formation are 1.53 mM/min and 0.33 mM/min for pig and cattle manure, respectively. Furthermore, our investigation shows that the maximum urease activity for pig feces occurs at approximately pH 7, and in cattle feces it is closer to pH 8, indicating that the predominant fecal ureolytic bacteria species differ between animal species. We believe that our study contributes to a better understanding of the urea hydrolysis process in manure and provides a basis for more accurate and animal-specific prediction models for urea hydrolysis rates and ammonia concentration in manures and thus can be used to predict ammonia volatilization rates from animal production.

## Introduction

The emission of ammonia (NH_3_) from agricultural systems is a major environmental problem. Most NH_3_ emissions come from animal production, especially from manure (a mixture of urine and feces). In addition, NH_3_ emission affects human and animal health [1]–[3]. NH_3_ in manure is formed by the hydrolysis of urinary urea (CO(NH_2_)_2_) and is catalyzed by microbial urease that is present in feces. The enzymatic decomposition of urea into carbonic acid (H_2_CO_3_) and volatile NH_3_ is initiated when urine and feces contact one another after being excreted. Reaction 1 represents the overall catalytic hydrolysis of urea, which enables organisms to use urea as a nitrogen source [4], [5]. The enzymatic hydrolysis of urea has a half-time of 20 ms at 25°C, and urease is among the most proficient known enzymes [6]–[8].

Reaction 1.
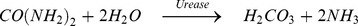



In aqueous solutions, the carbonic acid and NH_3_ generated from urea hydrolysis are in equilibrium with bicarbonate (HCO_3_^−^) and ammonium (NH_4_^+^) ions, respectively. Consequently, urea hydrolysis is associated with a subsequent increase in pH [4]. However, in the absence of active urease, urea is a very stable molecule with a half-time of approximately 40 years at 25°C [8], [9]. The non-catalytic decomposition of urea is not hydrolysis but proceeds through an elimination reaction to form isocyanate (HNCO) and NH_3_ (Reaction 2).

Reaction 2. 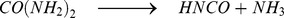



The NH_3_ emission level from manure depends on several factors including the animal species, urinary urea concentration, fecal urease activity, pH, temperature, manure management system, and air exchange rate. Therefore, NH_3_ production and emission can be reduced by altering the dietary composition, adding urease inhibitors, acidifying or cooling the manure, and modifying the house interior [2], [10]–[15]. To develop accurate prediction models for NH_3_ emission and efficient NH_3_ emission-reducing strategies for both pig and cattle production systems, it is necessary to understand the enzymatic process of NH_3_ formation in manure. However, accurate measurements of the urease activity in feces and manure from different animal species are still limited.

The aims of this study were to determine and compare the kinetics of urea hydrolysis as catalyzed by feces and manure from pigs and cattle and to make accurate estimates of kinetic parameters V_max_ and K′_m_. In addition, we determined the initial chemical and physical properties of feces, urine, and fresh manure and investigated the effects of pH on animal fecal urease activity. Our work shed light on the urea hydrolysis process in manure from pigs and cattle and has provided the basis for animal-specific prediction models of urea hydrolysis rates and NH_3_ concentrations in manures, and thus NH_3_ volatilization rates from animal production.

## Materials and Methods

Most chemicals and reagents were purchased from Sigma-Aldrich. Urea stock solutions (1 M and 4 M) were prepared by dissolving urea (Sigma 51459, puriss. p.a., ACS reagent, ≥99.5% (T)) in ultra pure water just before use. Phosphate buffer stock solutions (400 mM) were prepared by mixing phosphate salts NaH_2_PO_4_·H_2_O (Sigma S9638, ACS reagent, 98.0–102.0%) and Na_2_HPO_4_·7H_2_O (Sigma 30413, puriss. p.a., ACS reagent, ≥99%) in certain proportions to produce pH values of 6.0, 7.0, and 8.0 according to Ruzin [16]. In addition, citric acid-Na_2_HPO_4_-buffered stock solution (400 mM) pH 5.0 was prepared by mixing certain amounts of citric acid (Sigma 251275, ACS reagent, ≥99.5%) and Na_2_HPO_4_·7H_2_O. A 400 mM HEPES (Sigma H3375, ≥99.5%) buffer stock solution was titrated to pH 9.0 with 1 M NaOH. All stock solutions were prepared a few hours before each series of experiments. Concentrated (98%) sulfuric acid (100748, Merck KGaA, Germany), Kjeltab catalyst tablets (Thompson & Capper, UK), 32% sodium hydroxide (28225, VWR, Denmark), and boric acid (Sigma 31144) were used for the Kjeldahl analyses. A FOSS 2200 Kjeltec Auto Distillation apparatus was used for all distillations. A PHM210 pH meter with ±0.01 pH units of accuracy (Meterlab, Radiometer Analytical, Lyon, France) was used for all pH measurements. Ultra pure water from an Ultra Clear UV system (SG Water, Hamburg, Germany) was used in all experiments.

### Collecting Urine and Feces Samples

Fresh urine and feces samples were collected from fattening pigs (70–100 kg) and beef cows (500–600 kg). The pigs were approximately 3-5 months of age, and they were kept in an intensive housing system with a slatted floor. The animals were given wet feed made from wheat, barley, and soya beans that was fortified with minerals and vitamins; they had free access to water. The cattle were a cross between Danish Red and Simmental races at 4–6 years of age. The cattle were kept in a loose-housing system and were primarily fed clover-grass silage supplemented with compound feed for dairy cattle. Feces and urine samples from individual animals were collected separately in clean plastic bags to ensure that there was no mixing prior to the experiments. Both the feces and urine samples were grabbed directly upon excretion from the animals to prevent any contact with the barn floor. All the samples were stored at 4°C during transportation. Equal amounts of feces from five specimens were pooled for both pigs and cattle. In addition, equal amounts of urine from five animals were pooled and used in the experiments. Half the feces and urine pools were saved at −80°C for later use in chemical analyses and for determining the relative urease activity at different pH values. All urease activity measurements in fresh feces and manure were conducted within two days after sample collection. The urine and feces pools were stored at 4°C until use. However, the urease activity in thawed feces pools that had been saved at −80°C was measured for comparison.

### Ethics Statement

The urine and feces samples were collected by using a self-made “bucket on a stick” without touching the animals. The animals were never touched and were never stimulated or forced to excrete urine or feces. Because the animals experienced no “*pain, suffering, anxiety or lasting harm*”, approval from the Danish Inspectorate for Animal Experiments was not necessary according to the relevant Danish legislation (Bekendtgørelse af lov om dyreforsøg). The urine and feces samples used in this study were collected with permission from the animal owners.

### Chemical Analyses of Feces, Urine, and Fresh Manure

Three samples of pooled feces, pooled urine, and feces:urine mixtures (at a weight∶volume (w∶v) ratio of 1.0∶3.0 for pigs and 3.0∶2.0 for cattle) were analyzed for pH, dry matter, total Kjeldahl nitrogen (TKN  =  Organic-N + NH_3_-N + NH_4_^+^-N) concentration, total ammoniacal nitrogen (TAN  =  NH_3_-N + NH_4_^+^-N) concentration, and urea nitrogen (UN  =  Urea-N) concentration according to Table 1. Before the pH measurements of the feces, 10 g of fresh feces were thoroughly mixed with 30 ml of ultra pure water. For the dry matter determinations, fresh feces or manure samples were evaporated to dryness in an oven at 105°C for at least 24 h until the weights of the samples were constant. The TKN and TAN concentrations were determined by using 3 ml of urine or 2–3 g of feces or manure (samples were weighed before analysis) [17]–[19]. The initial urea concentration ([Urea]) in urine was calculated by subtracting the initial TAN concentration in urine [TAN_i,urine_] from the final TAN concentration [TAN_f,urine_] that was generated after the complete enzymatic hydrolysis of urea in urine by jack bean urease (Sigma 94282, activity ∼35 units/mg) and then multiplying this difference by 0.5 according to Eq. 1 because two NH_3_ molecules are generated from the hydrolysis of each urea molecule. For this determination, 56 ml of pooled urine was added to 4 ml of 400 mM phosphate buffer, pH 7.0 and 20 ml of jack bean urease solution (0.1 mg/ml equaling 3.5 units/ml) for a final concentration of 0.875 units/ml in the diluted urine solution to equal 1.25 units per ml of pure urine. The reaction mixture was incubated for 8 h at 25°C on a magnetic stirrer (mixing was performed during the first five minutes of incubation, and the reaction mixture was also stirred for 20 s at 300 rpm before each sampling). The TAN was determined after 5 min, 2 h, 4 h, 6 h, and 8 h of incubation, and at 8 h the reaction had reached completion. The final constant TAN reached upon the completion of the reaction was defined as the TAN_f,urine_ (Figure S1).

**Table 1:** The chemical and physical properties of feces, urine, and manure samples (Mean±SD; n = 3).

	Animal species	TKN		TAN		[UN]	[Urea]	Dry matter	pH
		(mmol/kg)	(mmol/l)	(mmol/kg)	(mmol/l)	(mmol/l)	(mmol/l)	(%)	
Feces	Pig	578.8±1.2	n.a^1^	39.6±4.6	n.a	n.a	n.a	15.32±0.09	6.89±0.01^2^
	Cattle	337.8±33.0	n.a	21.2±0.4	n.a.	n.a.	n.a.	11.44±0.22	7.02±0.02^3^
		P<0.001		P<0.01				P<0.001	P<0.001
Urine	Pig	n.a	350.2±2.1	n.a	23.6±1.0	198.4±5.0	99.2±2.5	1.86±0.84	7.69±0.03
	Cattle	n.a	261.3±0.9	n.a.	15.9±1.0	152.7±1.1	76.4±0.5	3.03±0.01	8.55±0.02
			P<0.001		P<0.001	P<0.001	P<0.001	P>0.05	P<0.001
Manure^4^	Pig	n.a	369.4±7.7	n.a	87.2±1.6	n.a.	n.a.	3.71±0.09	7.05±0.01
	Cattle	n.a	317.4±4.8	n.a	20.5±0.2	n.a	n.a	7.81±0.07	7.87±0.01
			P<0.001		P<0.001			P<0.001	P<0.001

The p-value obtained in each test of significance between the values for pigs and cattle is indicated below each pair of measurements. Thus, at a significance level of 0.05 all the measured properties are significantly different between pigs and cattle except the dry matter of urine (P>0.05).

1 n.a.: not available.

2 pH was measured in a mixture of 1∶3 (wt:v) feces and water.

3 pH was measured in a mixture of 3∶2 (wt:v) feces and water.

4 Pig manure was prepared by mixing feces and urine in a (wt:v)-ratio of 1∶3, and cattle manure was prepared by mixing feces and urine in a 3∶2 (wt:v)-ratio. TAN and pH were measured immediately after mixing the fresh feces and urine.



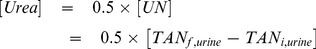
(1)


### Kinetic Measurements of Urease Activity in Feces

The amounts and ratios of feces and urine produced by animals depend on several factors including their diet and water supply [20], [21]. Some animal studies suggest that the (w∶v)-ratio of feces:urine produced by fattening pigs is approximately 1∶3 [20], [22] and that of cattle is approximately 3∶2 [21], [23]. Thus, to determine the kinetics of urease activity in pig feces, mixtures (approximately 40 ml of total volume) containing 10 g of pooled feces and 30 ml of urea-phosphate buffer solution, pH 7.0 with different urea concentrations were incubated in 50 ml beakers with magnetic stirring. For the kinetic measurements of urease activity in cattle feces, mixtures (containing approximately 30 ml of total volume) containing 18 g of pooled feces and 12 ml of urea-phosphate buffer solution, pH 7.0 with different urea concentrations were incubated in beakers while stirring. The stirring rate for all these kinetic experiments was 300 rpm during the 5 min incubation. Two to three hours before the kinetic measurements, the fecal samples and all solutions were placed in a water bath at a constant temperature of 25°C. The feces samples were subsequently prepared for the kinetic experiments; for example, to obtain a final urea concentration of 400 mM urea in a 40 ml reaction sample, 10 g of fecal sample was added to 23 ml of ultra pure water before being titrated to pH 7.0 with approximately 0.1–0.2 ml of 1 M NaOH. Afterwards, 3 ml of 400 mM phosphate buffer, pH 7.0 and 4 ml of 4.0 M stock urea solution were added. The final urea concentrations were 0.0 mM, 20 mM, 40 mM, 80 mM, 100 mM, 200 mM, 400 mM, and 600 mM for the experiments with fresh pig feces, and 0.0 mM, 10 mM, 20 mM, 40 mM, 60 mM, 80 mM, 120 mM, and 160 mM for those with fresh cattle feces. The same procedure was used for the thawed feces samples except that the final urea concentrations in the experiments were 0.0 mM, 2.0 mM, 4.0 mM, 8.0 mM, 20 mM, 40 mM, 60 mM, and 80 mM for both species. The urea hydrolysis reactions were initiated by adding the amounts of stock urea solution (1.0 or 4.0 M) corresponding to the desired final urea concentrations of the mixtures. The 1.0 M urea stock solution was used to prepare the reactions with 2.0–100 mM urea, and the 4.0 M urea stock solution was used for reactions containing 120–600 mM urea. For each substrate (urea) concentration, the amount of NH_3_ nitrogen generated during the 5 min reaction time was calculated by subtracting the initial amounts of ammoniacal nitrogen in feces and urea-buffer solutions from the final amount of ammoniacal nitrogen at the end of the reaction. Thus, for the kinetic measurements of urease activity in feces, 3 ml of sample was taken from each reaction mixture after reacting for 5 min and analyzed by Kjeldahl method to determine the TAN concentration [17]–[19]. Experiments showed that adding 75 ml of ultra pure water and 60 ml of 32% sodium hydroxide (NaOH) to the reactions as described in the Kjeldahl method [17]–[19] completely stops urease activity (there is no further increase in the TAN). Thus, no urea is hydrolyzed between the time of NaOH addition and the Kjeldahl distillation. To verify that the pH remained constant during the kinetic reaction, the pH of the mixture was measured throughout the whole reaction, from t = 0 min to t = 5 min. All experiments were performed in triplicate. The kinetics of urea hydrolysis by pig and cattle feces was characterized by determining the maximum reaction rate V_max_ and the apparent Michaelis constant K′_m_ according to Eq. 2 and Eq. 3.

### Measurements of Urease Activity in Fresh Manure

To make fresh manure, pooled feces and pooled urine samples from five specimens were mixed in (w∶v)-ratios of 1.0∶3.0 and 3.0∶2.0 for pigs and cattle, respectively. Thus, pig manure was made by mixing 20 g of pooled pig feces with 60 ml of pooled pig urine and cattle manure was made by mixing 60 g of pooled feces with 40 ml of pooled urine in 140 ml beakers. The fresh manure was then made homogenous by magnetic stirring at 300 rpm for 5 min before the beakers were covered with parafilm and incubated at 25°C. TAN concentration and pH of the manure samples were measured immediately after mixing (t∼0), homogenization (t = 5 min), and at incubation times of 30 min, 1 h, 2 h, 4 h, 6 h, 8 h, and up to approximately 100 h. The initial TAN of the manure (t = 0) was calculated by adding the determined TAN value of urine with that of feces. The TAN concentrations were determined by Kjeldahl method [17]–[19] and all experiments were performed in triplicate.

### Determining Fecal Urease Activity at Different pH Values

The fecal urease activity was determined under buffered conditions at pH values of 5.0, 6.0, 7.0, 8.0, and 9.0. Citric acid/Na_2_HPO_4_ buffer at a 40 mM final concentration was used in the mixture for pH 5.0, 40 mM phosphate buffers were used for pH 6.0, 7.0, and 8.0, and 40 mM HEPES was used as a buffer for pH 9.0. The temperatures of all samples and solutions were equilibrated in a water bath at 25°C before mixing. To directly compare the urease activity in feces from pigs and cattle, the same weights for feces and a 1.0∶3.0 (w∶v) ratio of feces:liquid were used for both species. According to the kinetic data, the rate of urea hydrolysis is close to a V_max_ at 0.2 M urea for both pig and cattle feces and, therefore, this urea concentration was used to determine the urease activity at different pH values. Thus, 10 g of pooled pig or cattle feces was mixed with 23 ml of ultra pure water in a 50 ml beaker and the pH was adjusted to the indicated pH value by adding sulfuric acid (1 M) or sodium hydroxide (1 M). Subsequently, 3 ml of 400 mM buffer stock solution (citric acid/Na_2_HPO_4_ buffer, phosphate buffer, or HEPES buffer) was added to keep the adjusted pH constant. The reaction was initiated by adding 4 ml of 2 M urea stock solution to a final concentration of 0.2 M and a total volume of 40 ml. The reactions were performed at 25°C while stirring at 300 rpm. After a reaction time of 5 min, the TAN concentration was determined [17]–[19]. The amount of ammoniacal nitrogen generated during the reaction was determined by subtracting the initial amounts of ammoniacal nitrogen present in feces and urea-buffer solutions. All the experiments were performed in triplicate.

### Enzyme Kinetics and Statistical Analyses

**Figure 1:**
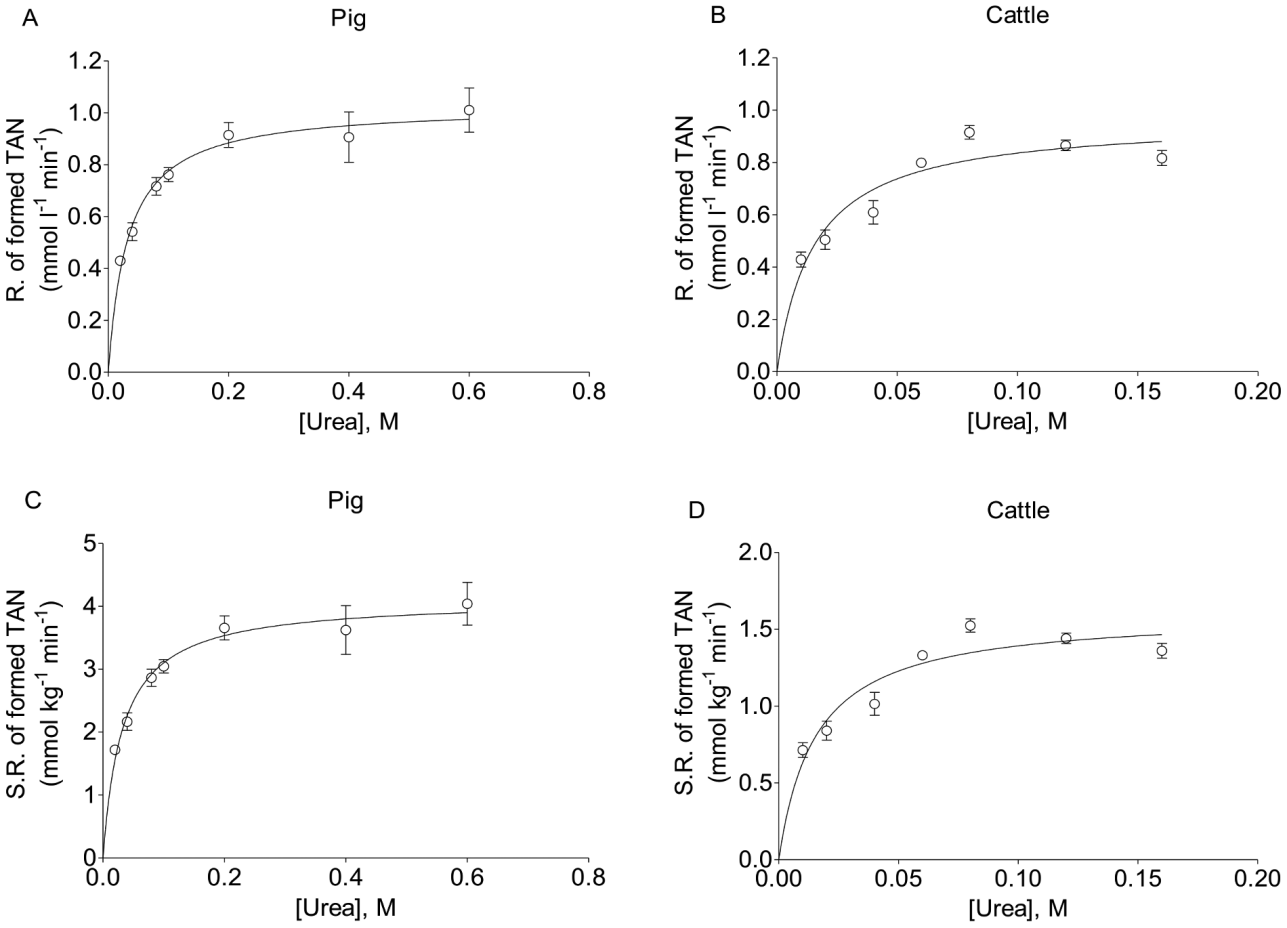
The rates of formed TAN as catalyzed by fresh pig and cattle feces. The rate of TAN formed (R. of formed TAN; panels A and B) and specific rate of TAN formed (S.R. of formed TAN; panels C and D) as catalyzed by pig feces (panels A and C) and cattle feces (panels B and D) in reaction mixtures containing fresh feces and different concentrations of urea.

**Figure 2:**
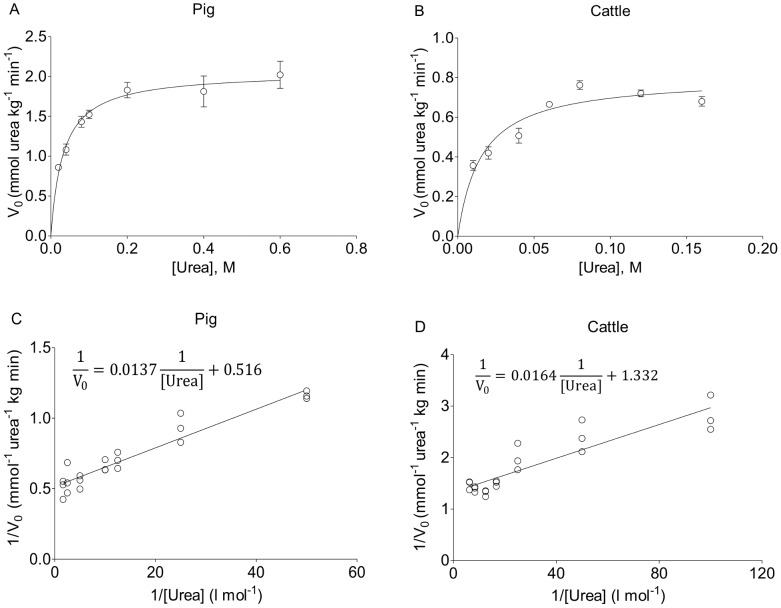
The Michaelis-Menten kinetics of the urease activity in fresh pig and cattle feces. Michaelis-Menten curves (panels A and B) and Lineweaver-Burk plots (panels C and D) for the specific reaction velocities of hydrolyzed urea (V_0_) as catalyzed by pig feces (panels A and C) and cattle feces (panels B and D). The curves are generated from Figure 1 data. The maximum specific V_max_ and K'_m_ values of the urease activity in fresh feces from pigs and cattle were determined from the graphic presentations. The goodness of fit values (R^2^) were 0.84 (panel A) and 0.91 (panel C) for the pig feces and 0.82 (panel B) and 0.81 (panel D) for the cattle feces.



Enzymatic reactions such as the hydrolysis of urea as catalyzed by urease can be described by Michaelis-Menten kinetics according to Eq. 2, where V is the rate of the enzymatic reaction, [S] is the substrate concentration, V_max_ is the maximum rate of the enzymatic reaction, and K′_m_ is the apparent Michaelis constant [24]. The data in Figure 1, Figure 2A, Figure 2B, Figure S3, Figure S4A, and Figure S4B were analyzed by using the Michaelis-Menten model.

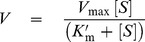
(2)By rearranging the Michaelis-Menten equation (Eq. 2) into the Lineweaver-Burk equation (Eq. 3), a linear regression of enzymatic reaction data 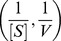 can be used to determine the V_max_ and K′_m_ values for the fecal urease activity in a Lineweaver-Burk plot [25]. The data in Figure 2C, Figure 2D, Figure S4C, and Figure S4D were analyzed according to the Lineweaver-Burk equation.
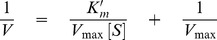
(3)


**Figure 3:**
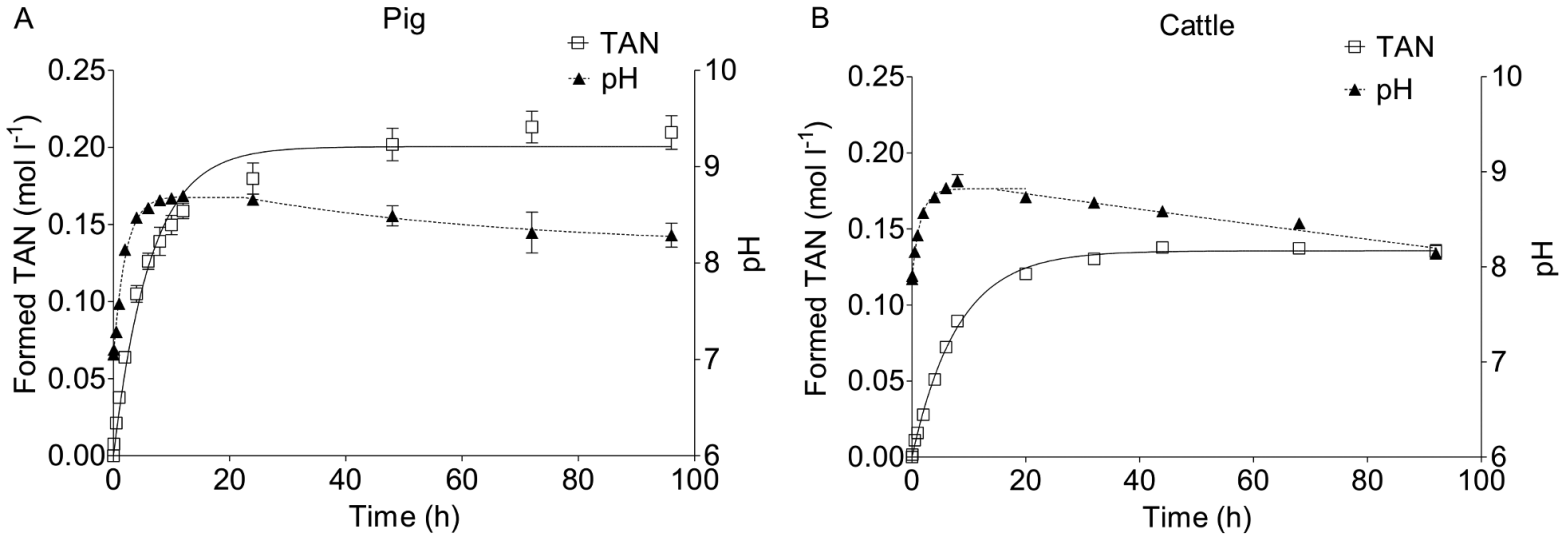
Urease activity in fresh manure from pigs and cattle. The formed TAN and changes in pH over time in fresh pig manure (panel A) and fresh cattle manure (panel B). During the first hours after mixing urine and feces, the concentration of formed TAN (open squares) and pH (filled triangles) increase rapidly in both pig and cattle manures. However, the rate of TAN formation in pig manure is significantly faster than it is in cattle manure and the TAN concentration reaches a higher plateau in pig manure than in cattle manure. In both manures, the pH decrease continuously after reaching a maximum.

A Student's t-test was used to determine if the nitrogen content, dry matter, and pH values for feces, urine, and manure samples are significantly different between pigs and cattle (Table 1), and to compare the urease kinetic values for V_max_ and K′_m_ between pig and cattle feces at a significance level of α = 0.05 (Table 2 and Table S1). A regression analysis by phase exponential association was used to determine the maximum TAN formation level as shown in Figure 3A, Figure 3B, and Figure S1. The pH change over time was determined by the one phase association and one phase decay regression in Figures 3A and 3B. All statistical analyses were performed with GraphPad Prism.

**Table 2:** Kinetic parameters of the urease activity in fresh feces.

Animal species	Temperature	*V_max_*	*K'_m_*	R^2^
	(°C)	(mmol urea/kg/min)	(mM)	Goodness of fit
Pig	25	2.06±0.08	32.59±5.65	0.84
Cattle	25	0.80±0.04	15.43±2.94	0.82
		P<0.001	P>0.05	

The *V_max_* and *K'_m_* values of fecal urease activity from pigs and cattle were determined by Michaelis-Menten kinetic analysis (Mean±S.E.).

## Results

### Comparing the Chemical and Physical Properties of Feces, Urine, and Fresh Manure from Pigs and Cattle

**Figure 4:**
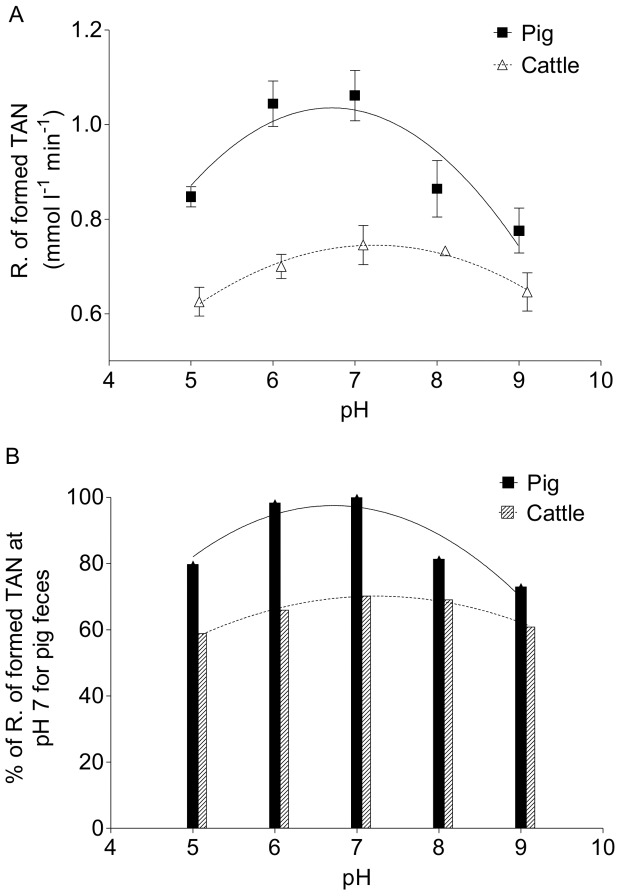
The effect of the pH on fecal urease activity. Urease activity at different pH values are presented as the rate of TAN formation (R. of formed TAN; panels A) and the relative R. of formed TAN compared with that of pig feces at pH 7 (panel B). The optimal pH for urea hydrolysis catalyzed by fecal urease is approximately pH 7 for pig feces and between pH 7 and 8 for cattle feces.

The initial properties including the TKN, TAN, and UN concentrations, dry matter, and pH of feces, urine, and fresh manure from pigs and cattle were determined (Table 1). All the TKN values were higher for the pig samples than for the corresponding cattle samples. Thus, the highest TKN concentration was found in pig feces with a value of 578.8±1.2 mmol/kg and that of cattle feces was only 337.8±33.0 mmol/kg (p<0.05). The TKN values for pig and cattle urine were 350.2±2.1 mM and 261.3±0.9 mM, respectively. In addition, the TAN measurements for pig feces (39.6±4.6 mmol/kg) and urine (23.6±1.0 mM) were significantly higher than the values for cattle feces (21.2±0.4 mmol/kg) and urine (15.9±1.0 mM), respectively. In addition, the urea concentrations in the urine samples were evaluated by finding the UN values. The urea concentration of pig urine (99.2±2.5 mM) was significantly higher than it was in cattle urine (76.4±0.5 mM). The dry matter of pig feces (15.32±0.09%) was approximately 4% higher than it was for cattle feces (11.44±0.22%), and the pH values of both pig feces (pH 6.89±0.01) and urine (pH 7.69±0.03) were lower than the corresponding values for cattle (pH 7.02±0.02 and 8.55±0.02, respectively) (p<0.05). With the exception of the TAN concentration in pig manure, all the values measured in fresh manure samples (combined feces and urine samples) were consistent with the expected values based on those determined for the separate feces and urine samples and their ratios in the combined feces and urine samples. The relatively high TAN concentration in pig manure (87.2±1.6 mM; Table 1) is most likely caused by the significantly faster formation of NH_3_ in manure from pigs than from cattle when feces and urine are mixed (Figure 3). Therefore, the initial TAN concentrations used to determine the TAN formed in the manure reactions (Figures 1, 3, 4, and Figures S2 and S3) were calculated by adding the proportions of TAN originating from pure feces and urine (or urea stock solution) (Table 1).

### Urease Activity in Feces from Pigs and Cattle

The kinetics of urea hydrolysis as catalyzed by fresh feces from pigs and cattle were investigated by first determining the rates of TAN formation in reaction mixtures containing feces and different urea concentrations (Figure 1). To obtain accurate enzymatic reaction velocities for the fecal samples, the rates of NH_3_ formation at different urea concentrations should be determined during the initial phase of the reactions and at a time when the levels of TAN formation are sufficient to achieve significant and reliable TAN measurements by Kjeldahl method. Therefore, to identify the optimal reaction time for the initial rate measurements, the levels of TAN formed at different reaction times (5 min, 11 min, and 20 min) were determined in mixtures of pig feces and 100 mM urea and the relation between the calculated rate of TAN formation and corresponding reaction time was investigated (Figure S2). The results clearly show that the calculated rate of TAN formation decreases significantly when the reaction time increases. Thus, the reaction rate calculated from the TAN formed at 5 min (0.45 mM/min) was significantly higher than the rates calculated at 11 min (0.31 mM/min) and 20 min (0.22 mM/min). Therefore, the initial rates of TAN formation were calculated from the TAN formed during the first 5 min of the reaction (Figures 1A and 1B). The maximum rates determined for TAN formation in reactions with pig feces and cattle feces using regression analyses were 1.03±0.04 mM/min (R^2^ = 0.84) and 0.99±0.05 mM/min (R^2^ = 0.82), respectively (Figures 1A and 1B). In addition, a comparison of the rates of TAN formation at different urea concentrations for the two feces samples reveals that the maximum rate of TAN formation is reached at a lower concentration for the cattle feces than for the pig feces (Figures 1A and 1B). This finding indicates that pig feces require higher concentrations of urea to reach the maximum reaction rate of TAN formation for the 5 min incubation. For comparison, the specific rates of TAN formation, that is, the reaction rates per wet weight of fresh feces, were calculated for all the urea concentrations (Figures 1C and 1D). The results show that pig feces are a much better catalyst for TAN formation than cattle feces (Figures 1C and 1D). Thus, the maximum specific rates of TAN formation for pig feces and cattle feces according to regression analyses were 4.11±0.17 mmol/kg/min (R^2^ = 0.84) and 1.61±0.07 mmol/kg/min (R^2^ = 0.82), respectively (Figures 1C and 1D). Based on the assumption that the hydrolysis of each urea molecule generates two molecules of NH_3_, the specific rates of TAN formation (mmol/kg/min) were converted into specific reaction velocities of hydrolyzed urea (V_0_; mmol urea/kg/min) and presented in Michaelis-Menten curves (Figures 2A and 2B) and Lineweaver-Burk plots (Figures 2C and 2D). From the Michaelis-Menten curves, the specific V_max_ and K′_m_ values of the urease activity in fresh feces from pigs and cattle were determined. The V_max_ was 2.06±0.08 mmol urea/kg/min and 0.80±0.04 mmol urea/kg/min for pig feces and cattle feces, respectively (Table 2). The K′_m_ was 32.59±5.65 mmol urea/l and 15.43±2.94 mmol urea/l for pig feces and cattle feces, respectively (Table 2). For comparison, the V_max_ and K'_m_ values were also determined from the Lineweaver-Burk plots (Figures 2C and 2D). Both the V_max_ (1.94 mmol urea/kg/min for pig feces and 0.75 mmol urea/kg/min for cattle feces) and K′_m_ (26.58 mmol urea/l for pig feces and 12.31 mmol urea/l for cattle feces) from the Lineweaver-Burk plots were consistent with those determined from the Michaelis-Menten curves. The urease activities in thawed pig and cattle feces pools that had been saved at −80°C were also evaluated by Michaelis-Menten kinetics (Figures S3 and S4), and their corresponding V_max_ and K′_m_ values were calculated from the Michaelis-Menten curves (Table S1). The V_max_ was 1.63±0.12 mmol urea/kg/min and 0.51±0.01 mmol urea/kg/min for the thawed pig feces and cattle feces, respectively. The K′_m_ was 12.84±3.03 mmol urea/l and 2.58±0.34 mmol urea/l for the thawed pig feces and cattle feces, respectively (Table S1). The V_max_ and K′_m_ values determined from Lineweaver-Burk plots (Figures S4C and S4D) were 1.43 mmol urea/kg/min and 9.86 mmol urea/l for the thawed pig feces, respectively, and those for thawed cattle feces were 0.53 mmol urea/kg/min and 3.08 mmol urea/l, respectively.

### Urease Activity in Fresh Manure from Pigs and Cattle

To investigate and compare the urease activity in fresh manure from pigs and cattle, fresh feces and urine were mixed in (w∶v)-ratios of 1.0∶3.0 and 3.0∶2.0 for pigs and cattle, respectively (Figure 3). The concentration of formed TAN and the pH increased rapidly in both types of manure. However, the rate of TAN formation in pig manure is significantly faster than it is in cattle manure. Thus, the initial velocities of TAN formation based on measurements taken at 5 min after mixing are 1.53 mM/min and 0.33 mM/min for pig and cattle manure, respectively. After approximately 30 hours, the formed TAN concentration for pig manure reaches a plateau of ∼0.2 M (0.20±0.003 M; K = 0.16, R^2^ = 0.980) and that of cattle manure reaches a plateau of ∼0.14 M (0.14±0.001 M; K = 0.12, R^2^ = 0.998) (Figure 3) as determined by regression analyses through one-phase exponential association. For both manures, the pH change was fitted with a one phase association (Figure 3; R^2^ = 0.99 for both pig and cattle, n = 30). The pH in cattle manure reaches a maximum of 8.91 after 6–8 hours, and a maximum of pH 8.70 for pig manure is obtained after reacting for 8–10 hours. This finding indicates that the pH of cattle manure changes by a total of 1.04 pH units from the initial pH of 7.87 (Table 1). For the pig manure, the pH changes by a total of 1.65 pH units from the initial pH of 7.05 (Table 1). After reaching the plateau, the pH values for both manure preparations decrease through one phase decay (Figure 3; R^2^ = 0.64 for pigs (n = 12), and R^2^ = 0.87 for cattle (n = 18)). The pH of pig manure decreases, with 0.41 units for the 12–96 hour time period, and the pH of cattle manure decreases 0.76 units in the 8–92 hour time period (Figure 3).

### The pH Effect on Urease Activity in Feces from Pigs and Cattle

For a direct comparison of the urease activity in pig and cattle feces at different pH values, all reactions in this experiment contained the same amount of feces. Therefore, the rate of urea hydrolysis was lower for cattle feces than for pig feces (Figure 4). The initial rates of TAN formation were within ranges of 0.78–1.06 mM/min and 0.63–0.75 mM/min for pig feces and cattle feces, respectively. For both species, the fecal urease activity varied significantly with the pH but the cattle feces is less affected by changes in pH (Figure 4). By comparison, the relative rates of TAN formation were calculated with reference to that catalyzed by pig feces at pH 7.0 (100%, Figure 4A). The relative reaction rates of TAN formation for the pig feces were 80%, 98%, 81%, and 73% at pH values of 5.0, 6.0, 8.0, and 9.0, respectively (Figure 4B). The relative rates of TAN formation for cattle feces compared with that for pig feces at pH 7.0 were 59%, 66%, 70%, 69%, and 61% at pH values of 5.0, 6.0, 7.0, 8.0, and 9.0, respectively (Figure 4B). Thus, the results suggest that the optimal pH for urea hydrolysis as catalyzed by fecal urease is approximately pH 7 for pig feces and between pH 7 and 8 for cattle feces.

## Discussion

To understand the process of NH_3_ formation in animal manure, we have determined the chemical and physical properties of feces, urine, and fresh manure and characterized the urease activity in fresh feces and manure from pigs and cattle.

### Pig Samples Contain Higher Levels of Nitrogen Compounds

The measured concentrations of TKN and TAN, and the pH values for feces, urine, and manure from pigs (Table 1) were consistent with previous results [20], [22], [26]. With regards to the urinary urea concentration and dry matter of feces and urine from pigs, our results were lower than those reported by Canh et al. [20]. The observed concentrations of TKN and TAN in urine and manure from cattle (Table 1) were consistent with nitrogen excretion values reported in some other studies [27]–[29]. In addition, the pH of the fresh manure is consistent with the values reported by those studies [28], [29]. However, the amount of urea in urine and the dry matter in manure from cattle in the present study are lower than those observed by Bristow et al. [27] and Burgos et al. [29]. The differences in dry matter levels compared with other studies are likely caused by variations in water consumption between animal facilities. Furthermore, several factors including the dietary protein content, feed composition, and volume of urine produced are known to affect the composition of nitrogen compounds and their concentrations in urine and feces and lead to large variations in TKN, TAN, and urea concentrations. The fact that all TKN, TAN, and urea measurements are higher for the pig samples than for the cattle samples (Table 1) most likely reflects that the pigs are given feedstuff with higher protein contents, which affects the nitrogen composition of urine and feces [30]. In particular, the TKN and TAN values in pig feces are 71% and 87% higher than the values for cattle feces, respectively. The higher TAN concentrations in pig feces and urine could be caused by a more ready conversion of organic nitrogen into ammoniacal nitrogen in the pig samples than in the cattle excreta. In addition, the dry matter of the pig manure is significantly lower than it is for cattle manure, which has also been reported in other studies [29], [30]. Our results also show that the pH values of feces, urine, and fresh manure from pigs are all lower than the values for cattle (Table 1).

### Pig Feces Have a Higher Specific Urease Activity than Cattle Feces

By using Michaelis-Menten kinetic analyses, we have determined the specific urease activity of fresh feces from pigs and cattle at 25°C. We first determined and compared the activities in feces-urea mixtures with feces:liquid ratios equaling those in authentic manure from pigs and cattle (Figure 1A and 1B). The maximum rates of TAN formation in the reaction mixtures are approximately 1 mM/min for both mixtures, and the urea concentration at half-maximum reaction rates of TAN formation are very different for the reactions. Thus, to further elucidate the results and make a thorough kinetic comparison of the pig and cattle fecal urease activities, the kinetic data were converted into specific reaction velocities of hydrolyzed urea (mmol urea hydrolyzed per kg wet feces per min, Figure 2). The kinetic analyses showed that the maximum specific urease activity and the K'_m_ value are more than 2-fold higher for pig feces than for cattle feces. In kinetic analyses employing pure enzyme preparations, the Michaelis constant is an inverse measure of the affinity between the substrate and enzyme. Thus, the smaller the K_m_ value, the higher the affinity [24], [25]. However, with a complex biological material such as feces, the Michaelis constant of the urease activity is actually a measure of the “overall affinity” between urea and the microbial community in feces and depends on factors such as diffusion, membrane-spanning urea transporter characteristics, the urease enzyme, and other components of the urease system [4], [31]–[33]. Most microbial ureases are intracellular and, therefore, the urea must first reach the cells in feces and then be transported across the cytoplasmic membrane before it is degraded by urease. Thus, the fact that the K′_m_ value for pig feces (32.59±5.65 mM) is approximately two times higher than it is for cattle feces (15.43±2.94 mM) suggests that the “overall affinity” of urea is lower for pig feces than for cattle feces. This finding signifies that a lower urea concentration is required to saturate the urea hydrolysis capacity of cattle feces than that of pig feces. The differences between the fecal urease kinetic parameters of pigs and cattle may indicate that their feces are dominated by different ureolytic bacteria species.

Muck R.E. [19] previously determined the V_max_ (1.17±0.19 mg urea-N/g wet feces/h) and K_m_ (0.48±0.04 mg urea-N/g mixture) for bovine feces at 24°C. When converted into molar concentrations, these values roughly equal V_max_ and K_m_ values of 0.7±0.1 mmol urea/kg/min and 17.1±1.4 mmol urea/l, respectively. Thus, the kinetic parameters for cattle in our study are slightly different from those determined by Muck R.E. In contrast to the findings of Muck R.E., who used a 1 h incubation time in the urease kinetic experiments, we used a much shorter reaction time (5 min), which should give more correct initial reaction velocity measurements according to enzyme kinetic theory, and thus better V_max_ and K_m_ determinations. In addition, other researchers have previously used a value of 2 mM (2 µmol/g) for the Michaelis constant in studies of both pig and dairy-cow houses [15], [34], [35].

### Faster NH_3_ Production in Pig Manure than in Cattle Manure

The difference in the enzymatic reaction velocity of urea hydrolysis between pig and cattle feces was even more significant in authentic fresh manure when the ammoniacal nitrogen production was recorded (Figure 3). Thus, the initial velocity of TAN formation was more than 4-fold higher in fresh pig manure (1.53 mM/min) than in cattle manure (0.33 mM/min) despite the higher feces-to-urine ratio in cattle manure. That observation may be explained by factors affecting the urease activity including the different chemical composition, pH, dry matter (Table 1), and texture of pig and cattle manure and the higher concentration of urea in pig manure. According to the measured concentrations of urea in urine (Table 1) and the ratios of feces and urine in the manures, the initial urea concentrations in manure from pigs and cattle are approximately 75 mM and 30 mM, respectively. The lower rate of pH change in pig manure than in cattle manure after reaching the maximum pH (Figure 3) suggests that less NH_3_ vaporizes from the pig manure or/and that pig manure has a stronger buffer capacity than cattle manure close to the maximum pH.

### The Effects of the pH on the Fecal Urease Activity Suggest There Are Different Bacterial Communities in Feces from Pigs and Cattle

Our measurements of urea hydrolysis activity at different pH values show that the maximum urease activity for pig feces is observed at approximately pH 7, and that of cattle feces is closer to pH 8 (Figure 4). It is noteworthy that fresh pig manure has an initial pH of 7.05 and that of cattle manure is 7.87 (Table 1), which suggests that the bacterial communities in the feces from the two animal species have urease enzymes that are most efficient at the initial pH of the manure. Thus, these results indicate that the predominant ureolytic bacterial species responsible for the urea hydrolysis activity in feces are different between pigs and cattle and are adapted to species-specific conditions in the animal manures.

### Implications for NH_3_ Production and Volatilization from Manure

Our results show that TAN production is both significantly faster and higher in pig manure than in cattle manure, which is important in relation to the volatilization of NH_3_ from the two manure types. The rate of NH_3_ volatilization from manure is related to different factors including, for example, the urease enzyme activity, the equilibrium between NH_3_ and ammonium, the pH, the temperature, and the air velocity at the manure surface. Consequently, reducing the urea hydrolysis activity in manure by adding urease inhibitors, for example, will lead to a reduction in the NH_3_ production and volatilization levels as reported by Varel V.H. and colleagues [12], [36]. In Denmark, acidifying manure to pH <6 is an approved and established technology to reduce the volatilization of NH_3_ from animal production [14]. Our observations show that the acidification of both pig and cattle manure to pH 5–6 slightly reduces the urease activity (at a reduction of up to 10–20%) compared with the maximum activity observed at the optimal pH values (Figure 4). A previous study showed that the microbial activity as expressed by oxygen consumption, methanogenesis, and sulfate reduction in a slurry acidified to pH 5.5 was greatly reduced relative to that of untreated slurry [37]. Together, these observations show that some metabolic processes including NH_3_ formation from urea hydrolysis are almost unaffected and others are dramatically reduced or absent in acidified manure relative to normal manure.

The kinetic parameters of urease activity in feces and manure have been incorporated into the calculations and process modeling of NH_3_ concentration and volatilization from manure stores and animal houses in many studies [15], [19], [34], [35], [38]. We believe that the kinetic measurement and characterization of fecal urease activity for both pigs and cattle as presented in the current study will be useful in future studies to make more accurate and animal-specific prediction models for urea hydrolysis rates and NH_3_ concentrations in pig and cattle manures and thus, for NH_3_ volatilization rates from animal production.

## Supporting Information

Figure S1 **Determining the urea nitrogen concentration [UN] in urine.** Jack bean urease was added to the urine samples for urea hydrolysis. The TAN concentration was measured at different time points and the corresponding level of formed TAN was calculated by subtracting the initial TAN (TAN_i,urine_) concentration from the measured TAN (TAN_m,urine_) concentration. The final constant TAN reached at the completion of the reaction was defined as TAN_f,urine_. The final concentration of formed TAN (TAN_f,urine_- TAN_i,urine_) reached at the completion of the reaction equals [UN] and was used to calculate the initial urea concentration in urine.
(TIF)

Figure S2 **The relation between the reaction time and the rate of formed TAN.** Formed TAN (filled triangles) and the corresponding rate of formed TAN (R. of formed TAN; open squares) after different reaction times. The levels of formed TAN after 5 min, 11 min, and 20 min of reaction time were measured in mixtures containing pig feces and 100 mM urea. The highest R. of formed TAN is observed at a reaction time of 5 min.
(TIF)

Figure S3 **Rates of formed TAN as catalyzed by thawed pig and cattle feces.** The rate of TAN formation (R. of formed TAN; panels A and B) and the specific rate of TAN formation (S.R. of formed TAN; panels C and D) as catalyzed by thawed pig feces (panels A and C) and thawed cattle feces (panels B and D).
(TIF)

Figure S4 **The Michaelis-Menten kinetics of urease activity in thawed pig and cattle feces.** Michaelis-Menten curves (panels A and B) and Lineweaver-Burk plots (panels C and D) for the specific reaction velocities of hydrolyzed urea (V_0_) as catalyzed by thawed pig feces (panels A and C) and thawed cattle feces (panels B and D). The curves are generated from Figure S3 data. The goodness of fit values (R^2^) were 0.89 (panel A) and 0.86 (panel C) for the pig feces and 0.90 (panel B) and 0.93 (panel D) for cattle feces.
(TIF)

Table S1 **Kinetic parameters of the urease activity in thawed feces.** *V_max_* and *K'_m_* values of the urease activity of thawed feces from pig and cattle were determined by Michaelis-Menten kinetic analysis (Mean±S.E.).
(DOCX)

## References

[1] Aneja VP, Schlesinger WH, Erisman JW (2009) Effects of agriculture upon the air quality and climate: research, policy, and regulations. Environ Sci Technol 43: 4234–4240. 19603628 10.1021/es8024403

[2] Bouwman AF, VanderHoek KW (1997) Scenarios of animal waste production and fertilizer use and associated ammonia emission for the developing countries. Atmospheric Environment 31: 4095–4102.

[3] Eghball B, Power JF (1994) Beef-Cattle Feedlot Manure Management. Journal of Soil and Water Conservation 49: 113–122.

[4] Mobley HL, Island MD, Hausinger RP (1995) Molecular biology of microbial ureases. Microbiol Rev 59: 451–480. 7565414 10.1128/mr.59.3.451-480.1995 PMC239369

[5] Krajewska B (2009) Ureases I. Functional, catalytic and kinetic properties: A review. Journal of Molecular Catalysis B: Enzymatic 59: 9–21.

[6] Estiu G, Merz KM Jr (2004) The hydrolysis of urea and the proficiency of urease. J Am Chem Soc 126: 6932–6944. 15174863 10.1021/ja049327g

[7] Laidler KJ, Hoare JP (1950) The Molecular Kinetics of the Urea-Urease System. III. Heats and Entropies of Complex Formation and Reaction Journal of the American Chemical Society 72: 2489–2494.

[8] Callahan BP, Yuan Y, Wolfenden R (2005) The burden borne by urease. J Am Chem Soc 127: 10828–10829. 16076178 10.1021/ja0525399

[9] Shaw WHR, Bordeaux JJ (1955) The Decomposition of Urea in Aqueous Media. Journal of the American Chemical Society 77: 4729–4733.

[10] Voorburg JH, Kroodsma W (1992) Volatile Emissions of Housing Systems for Cattle. Livestock Production Science 31: 57–70.

[11] Vanderholm DH (1979) Handling of Manure from Different Livestock and Management-Systems. Journal of Animal Science 48: 113–120.

[12] Varel VH (1997) Use of urease inhibitors to control nitrogen loss from livestock waste. Bioresource Technology 62: 11–17.

[13] Stevens RJ, Laughlin RJ, Frost JP (1989) Effect of Acidification with Sulfuric-Acid on the Volatilization of Ammonia from Cow and Pig Slurries. Journal of Agricultural Science 113: 389–395.

[14] Kai P, Pedersen P, Jensen JE, Hansen MN, Sommer SG (2008) A whole-farm assessment of the efficacy of slurry acidification in reducing ammonia emissions. European Journal of Agronomy 28: 148–154.

[15] Aarnink AJA, Elzing A (1998) Dynamic model for ammonia volatilization in housing with partially slatted floors, for fattening pigs. Livestock Production Science 53: 153–169.

[16] Ruzin SE (1999) Plant Microtechnique and Microscopy. New York: Oxford University Press.

[17] Kjeldahl JZ (1883) A new method for the determination of nitrogen in organic bodies. Analytical Chemistry 22: 366.

[18] Lynch JM, Barbano DM (1999) Kjeldahl nitrogen analysis as a reference method for protein determination in dairy products. Journal of Aoac International 82: 1389–1398. 10589493

[19] Muck RE (1982) Urease Activity in Bovine Feces. Journal of Dairy Science 65: 2157–2163.

[20] Canh TT, Verstegen MW, Aarnink AJ, Schrama JW (1997) Influence of dietary factors on nitrogen partitioning and composition of urine and feces of fattening pigs. J Anim Sci 75: 700–706. 9078486 10.2527/1997.753700x

[21] ASABE (2005) Manure Production and Characteristics. ASABE Standard D384.2. American Society of Agricultural and Biological Engineers, St. Joseph, MI, USA.

[22] Canh TT, Aarnink AJA, Schutte JB, Sutton A, Langhout DJ, et al (1998) Dietary protein affects nitrogen excretion and ammonia emission from slurry of growing-finishing pigs. Livestock Production Science 56: 181–191.

[23] Morse D, Nordstedt RA, Head HH, Vanhorn HH (1994) Production and Characteristics of Manure from Lactating Dairy-Cows in Florida. Transactions of the Asae 37: 275–279.

[24] Michaelis L, Menten ML (1913) The kinetics of the inversion effect. Biochemische Zeitschrift 49: 333–369.

[25] Lineweaver H, Burk D (1934) The determination of enzyme dissociation constants. Journal of the American Chemical Society 56: 658–666.

[26] Sanchez M, Gonzalez JL (2005) The fertilizer value of pig slurry. I. Values depending on the type of operation. Bioresour Technol 96: 1117–1123. 15683901 10.1016/j.biortech.2004.10.002

[27] Bristow AW, Whitehead DC, Cockburn JE (1992) Nitrogenous Constituents in the Urine of Cattle, Sheep and Goats. Journal of the Science of Food and Agriculture 59: 387–394.

[28] Kulling DR, Menzi H, Krober TF, Neftel A, Sutter F, et al (2001) Emissions of ammonia, nitrous oxide and methane from different types of dairy manure during storage as affected by dietary protein content. Journal of Agricultural Science 137: 235–250.

[29] Burgos SA, Embertson NM, Zhao Y, Mitloehner FM, DePeters EJ, et al (2010) Prediction of ammonia emission from dairy cattle manure based on milk urea nitrogen: Relation of milk urea nitrogen to ammonia emissions. Journal of Dairy Science 93: 2377–2386. 20494146 10.3168/jds.2009-2415

[30] Portejoie S, Dourmad JY, Martinez J, Lebreton Y (2004) Effect of lowering dietary crude protein on nitrogen excretion, manure composition and ammonia emission from fattening pigs. Livestock Production Science 91: 45–55.

[31] Scow KM, Alexander M (1992) Effect of diffusion on the kinetics of biodegradation - experimental results with synthetic aggregates. Soil Science Society of America Journal 56: 128–134.

[32] Hopkins DW, Shiel RS (1996) Size and activity of soil microbial communities in long-term experimental grassland plots treated with manure and inorganic fertilizers. Biol Fertil Soils 22: 66–70.

[33] Button DK (1991) Biochemical basis for whole-cell uptake kinetics: specific affinity, oligotrophic capacity, and the meaning of the michaelis constant. Appl Environ Microbiol 57: 2033–2038. 16348524 10.1128/aem.57.7.2033-2038.1991 PMC183517

[34] Cortus EL, Lemay SP, Barber EM, Hill GA, Godbout S (2008) A dynamic model of ammonia emission from urine puddles. Biosystems Engineering 99: 390–402.

[35] Elzing A, Monteny GJ (1997) Modeling and experimental determination of ammonia emissions rates from a scale model dairy-cow house. Transactions of the Asae 40: 721–726.

[36] Varel VH, Robinson IM, Pond WG (1987) Effect of dietary copper sulfate, Aureo SP250, or clinoptilolite on ureolytic bacteria found in the pig large intestine. Appl Environ Microbiol 53: 2009–2012. 2823707 10.1128/aem.53.9.2009-2012.1987 PMC204049

[37] Ottosen LDM, Poulsen HV, Nielsen DA, Finster K, Nielsen LP, et al (2009) Observations on microbial activity in acidified pig slurry. Biosystems Engineering 102: 291–297.

[38] Cortus EL, Lemay SP, Barber EM (2010) Dynamic Simulation of Ammonia Concentration and Emission within Swine Barns: Part I. Model Development. Transactions of the Asabe 53: 911–923.

