# Supplementary figures and images for: A Determination and Comparison of Urease Activity in Feces and Fresh Manure from Pig and Cattle in Relation to Ammonia Production and pH Changes
Source: PLoS One. 2014 Nov 14;9(11):e110402. doi: 10.1371/journal.pone.0110402 (PMC4232307; doi:10.1371/journal.pone.0110402)

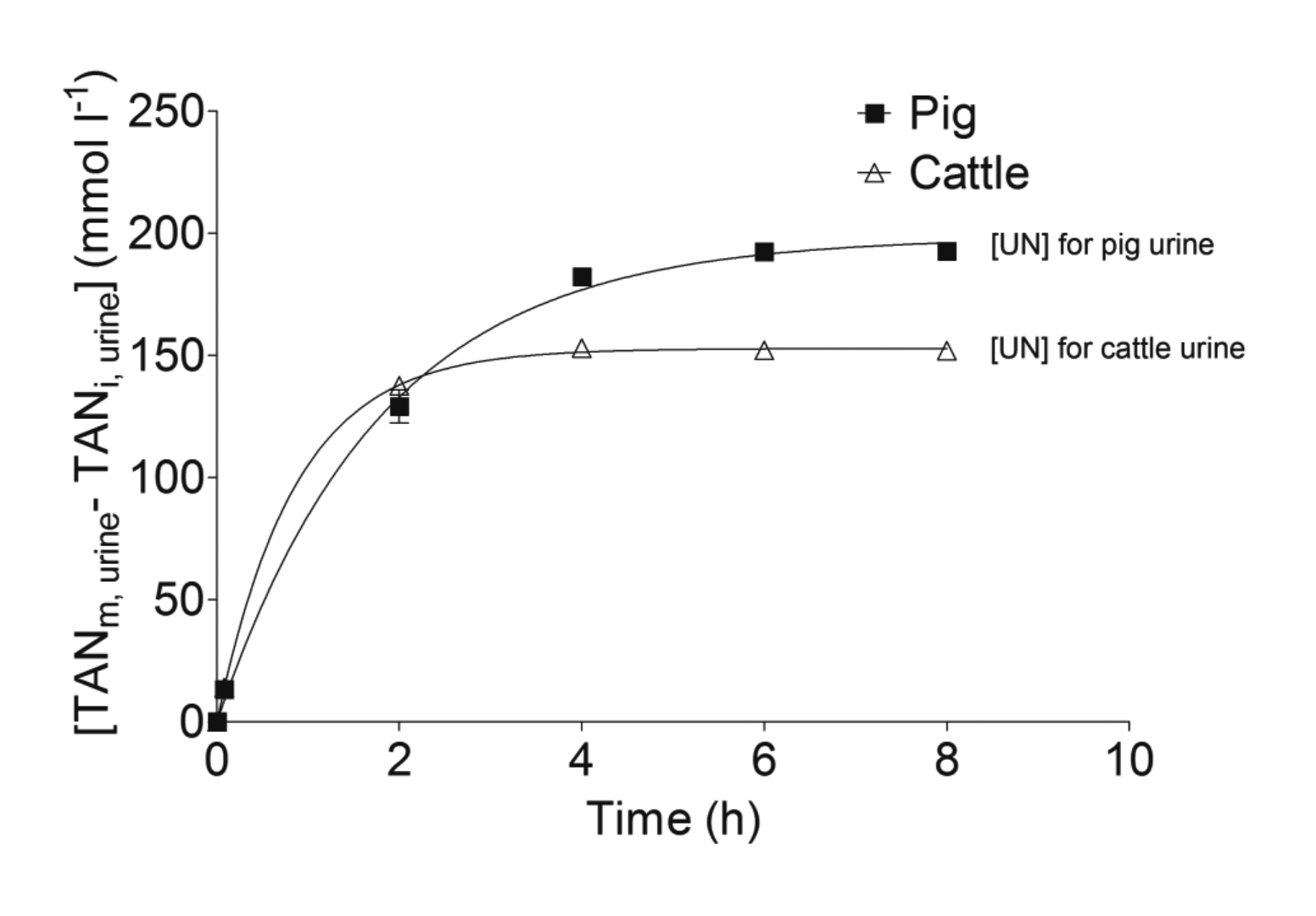

Supplement: Figure S1 — Determining the urea nitrogen concentration [UN] in urine. Jack bean urease was added to the urine samples for urea hydrolysis. The TAN concentration was measured at different time points and the corresponding level of formed TAN was calculated by subtracting the initial TAN (TANi,urine) concentration from the measured TAN (TANm,urine) concentration. The final constant TAN reached at the completion of the reaction was defined as TANf,urine. The final concentration of formed TAN (TANf,urine- TANi,urine) reached at the completion of the reaction equals [UN] and was used to calculate the initial urea concentration in urine. (TIF) [file pone.0110402.s001.tif]

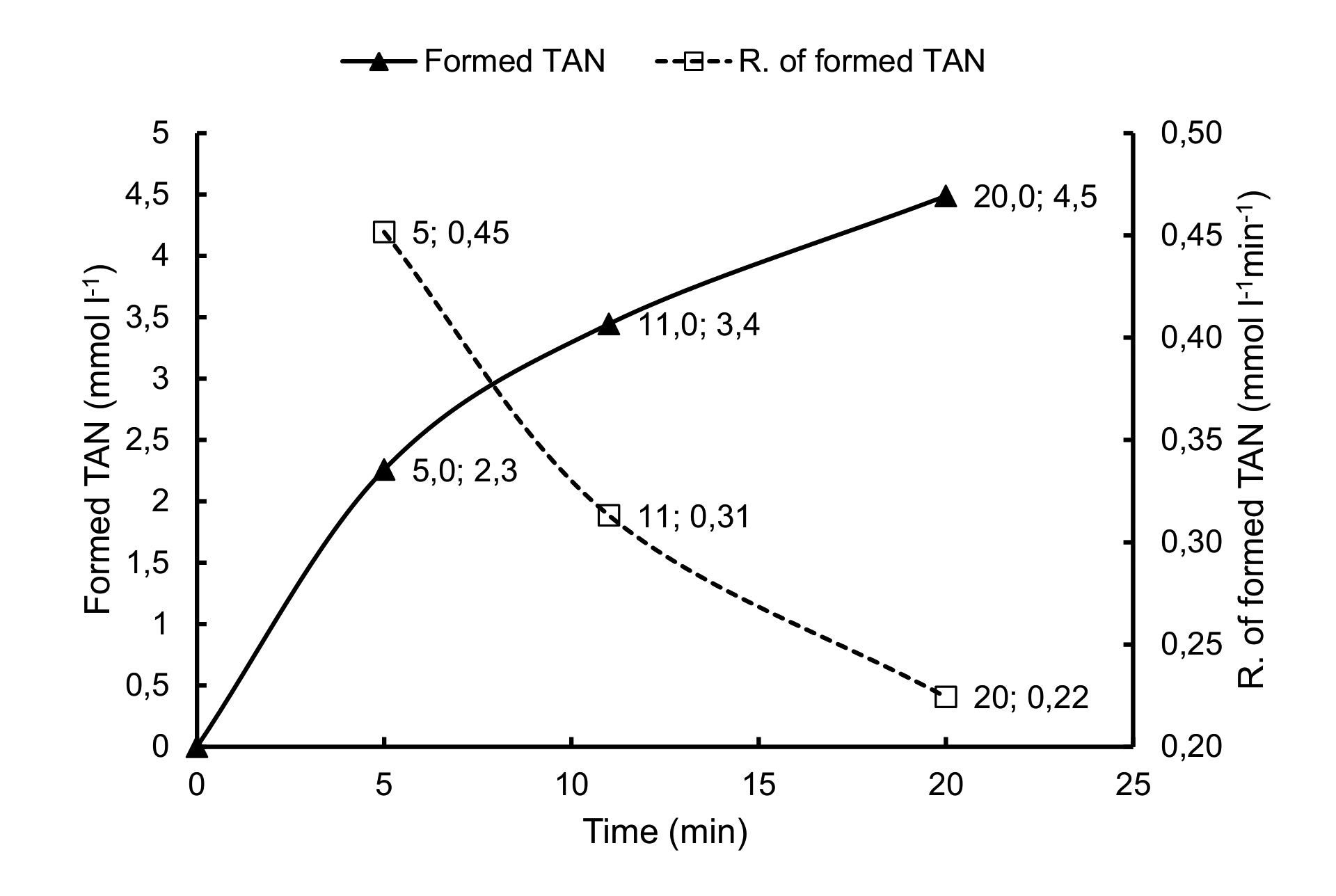

Supplement: Figure S2 — The relation between the reaction time and the rate of formed TAN. Formed TAN (filled triangles) and the corresponding rate of formed TAN (R. of formed TAN; open squares) after different reaction times. The levels of formed TAN after 5 min, 11 min, and 20 min of reaction time were measured in mixtures containing pig feces and 100 mM urea. The highest R. of formed TAN is observed at a reaction time of 5 min. (TIF) [file pone.0110402.s002.tif]

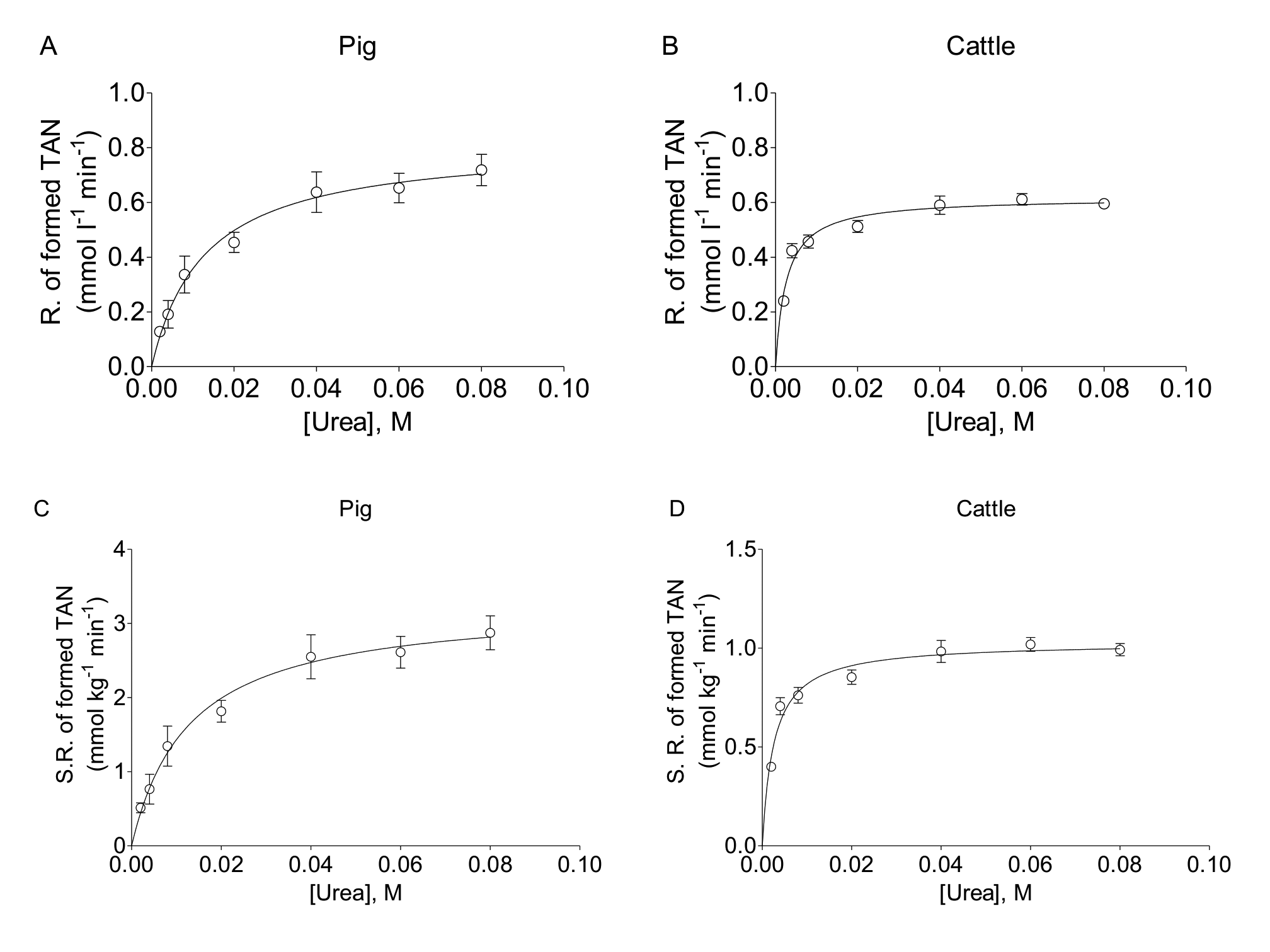

Supplement: Figure S3 — Rates of formed TAN as catalyzed by thawed pig and cattle feces. The rate of TAN formation (R. of formed TAN; panels A and B) and the specific rate of TAN formation (S.R. of formed TAN; panels C and D) as catalyzed by thawed pig feces (panels A and C) and thawed cattle feces (panels B and D). (TIF) [file pone.0110402.s003.tif]

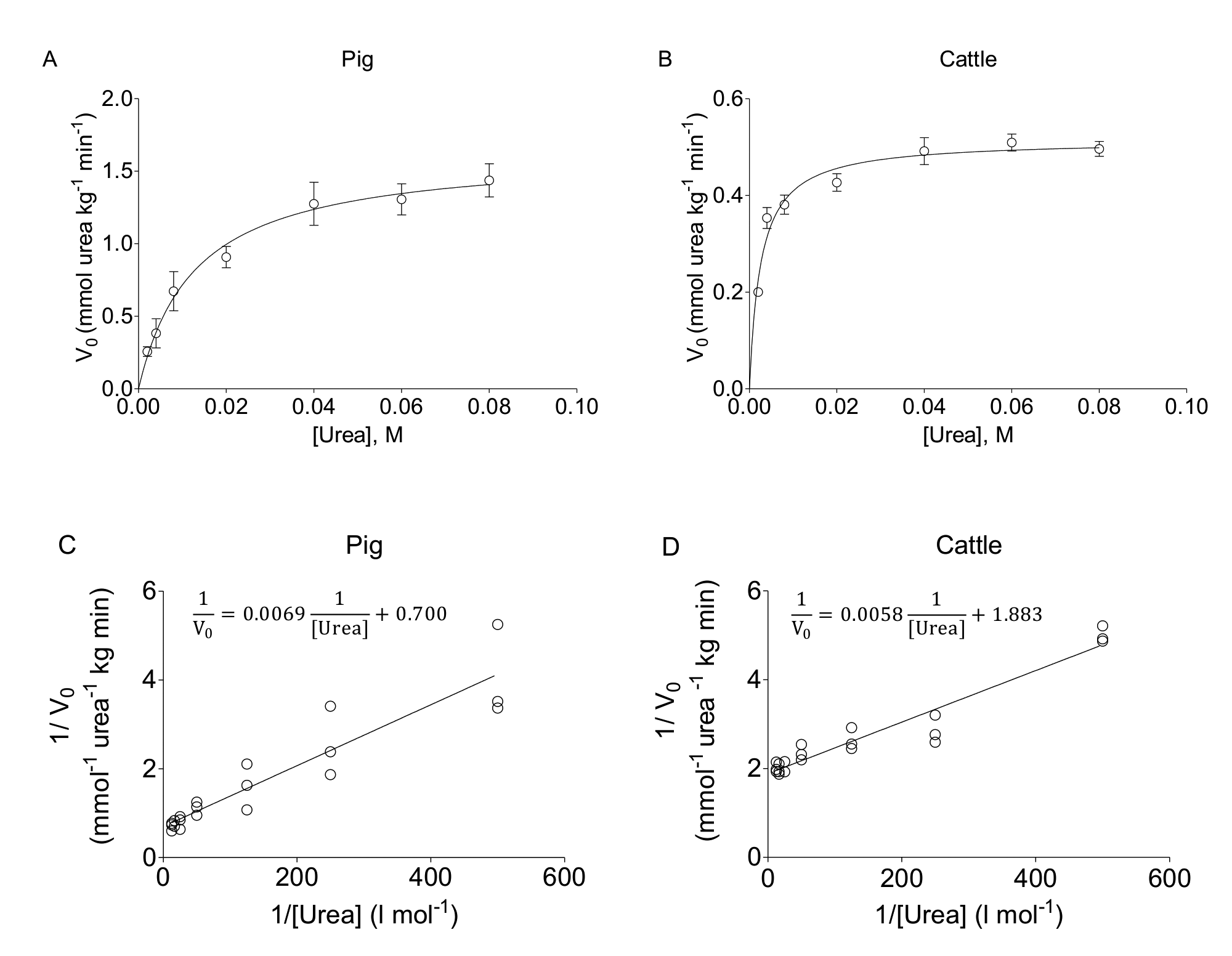

Supplement: Figure S4 — The Michaelis-Menten kinetics of urease activity in thawed pig and cattle feces. Michaelis-Menten curves (panels A and B) and Lineweaver-Burk plots (panels C and D) for the specific reaction velocities of hydrolyzed urea (V0) as catalyzed by thawed pig feces (panels A and C) and thawed cattle feces (panels B and D). The curves are generated from Figure S3 data. The goodness of fit values (R2) were 0.89 (panel A) and 0.86 (panel C) for the pig feces and 0.90 (panel B) and 0.93 (panel D) for cattle feces. (TIF) [file pone.0110402.s004.tif]
