# Supplementary material for: A Determination and Comparison of Urease Activity in Feces and Fresh Manure from Pig and Cattle in Relation to Ammonia Production and pH Changes
Source: PLoS One. 2014 Nov 14;9(11):e110402. doi: 10.1371/journal.pone.0110402 (PMC4232307; doi:10.1371/journal.pone.0110402)
Supplement: Table S1 — Kinetic parameters of the urease activity in thawed feces. Vmax and K'm values of the urease activity of thawed feces from pig and cattle were determined by Michaelis-Menten kinetic analysis (Mean±S.E.). (DOCX) [file pone.0110402.s005.docx]

| **Table S1. Kinetic parameters of the urease activity in thawed feces.** The *V_max_* and *K'_m_* values of the urease activity for the thawed feces from pig and cattle were determined by Michaelis-Menten kinetic analysis (Mean±S.E.). | | | | |
| --- | --- | --- | --- | --- |
| Animal species | Temperature | *V_max_* | *K'_m_* | R^2^ |
|  | (°C) | (mmol urea/kg/min) | (mM) | Goodness of fit |
| Pig | 25 | 1.63±0.12 | 12.84±3.03 | 0.89 |
| Cattle | 25 | 0.51±0.01 | 2.58±0.34 | 0.90 |
|  |  | P < 0.001 | P < 0.01 |  |
